# Supplementary figures and images for: Automated guided vehicle (AGV) path optimization method based on improved rapidly-exploring random trees
Source: PeerJ Comput Sci. 2025 Jun 18;11:e2915. doi: 10.7717/peerj-cs.2915 (PMC12192648; doi:10.7717/peerj-cs.2915)

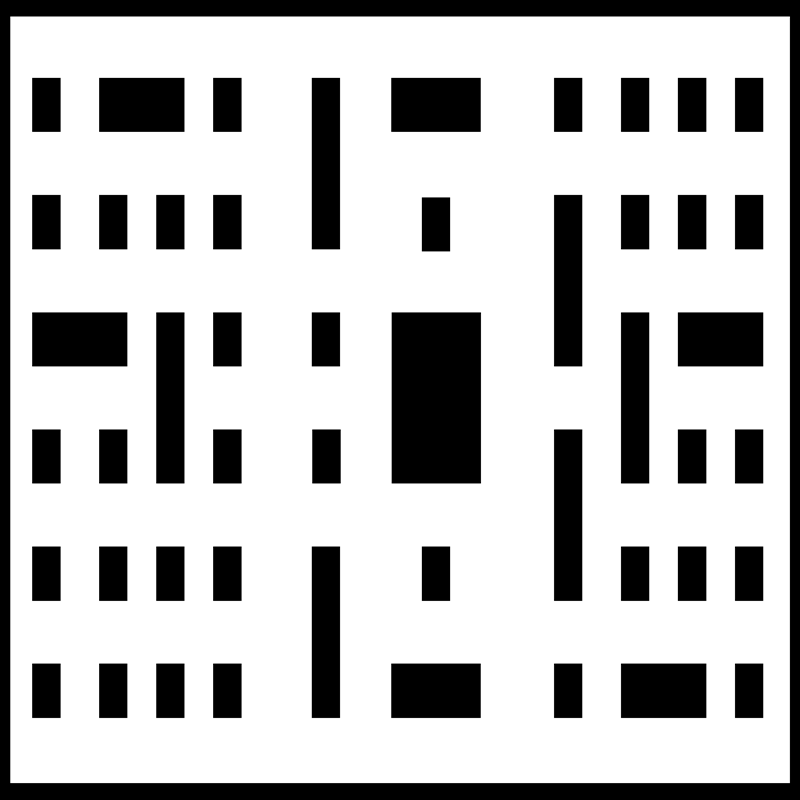

Supplement: Supplemental Information 2 [file peerj-cs-11-2915-s002.zip › dataset/new.png]

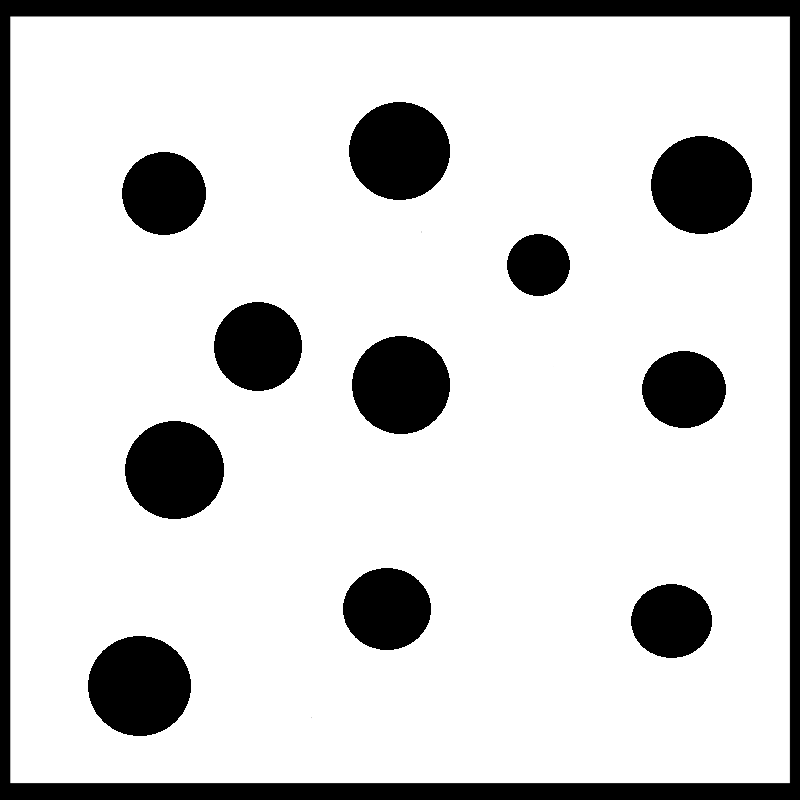

Supplement: Supplemental Information 2 [file peerj-cs-11-2915-s002.zip › dataset/new1.png]

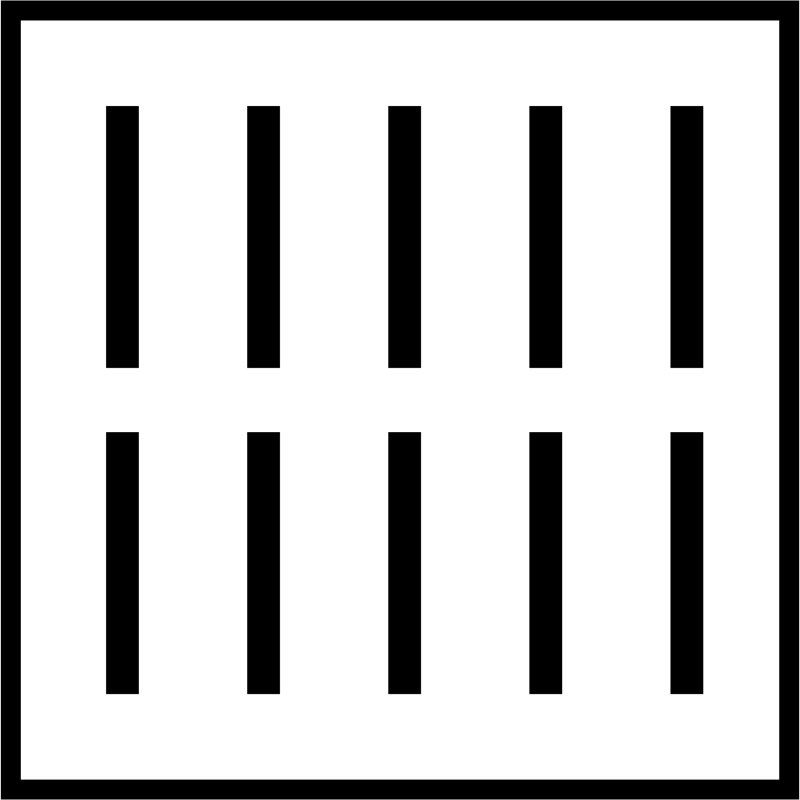

Supplement: Supplemental Information 2 [file peerj-cs-11-2915-s002.zip › dataset/new3.png]
